# Microbiological and Molecular Study of Paranasal Sinus Infections of Children with Malignancy and Unknown Origin Fever in Markazi Province, Iran

**DOI:** 10.1016/j.curtheres.2024.100745

**Published:** 2024-03-19

**Authors:** Kazem Ghaffari, Vahid Falahati, Tahereh Motallebirad, Mahdi Safarabadi, Amir Hossein Tashakor, Davood Azadi

**Affiliations:** 1 Department of Basic Sciences, Khomein University of Medical Sciences, Khomein, Iran; 2 Department of Pediatrics, Arak University of Medical Sciences, Arak, Iran; 3 Department of Nursing, Khomein University of Medical Sciences, Khomein, Iran; 4 Department of Microbiology, School of Medicine, Isfahan University of Medical Sciences, Isfahan, Iran; 5 Student Research Comittee, Khomein University Of Medical Sciences, Khomein, Iran

**Keywords:** 16S rRNA, Malignancy, Unknown origin fever

## Abstract

**Background:**

Children with malignancies are vulnerable to various infections, including sinus infections. Sinusitis is primarily caused by bacterial infections, followed by fungal infections. Due to this, evaluating the occurrence, diversity, and antibiotic susceptibility patterns of bacterial species that cause paranasal sinus infections in children with malignancy and unexplained fever is important.

**Objective:**

To investigate the bacterial species accountable for sinusitis in children with malignancy and unexplained fever, and determine their susceptibility to antibiotics.

**Methods:**

The study involved collecting 90 sinus samples from children aged 5 to 15 years with malignancy in Arak City, Iran. The isolates were identified using a combination of phenotypic, biochemical, and molecular techniques, including specific polymerase chain reaction and 16S ribosomal RNA gene sequencing. Drug susceptibility testing was performed following the Clinical & Laboratory Standards Institute 2021 guidelines.

**Results:**

A total of 36 isolates (40%) were obtained, including 4 isolates of *Nocardia* (11.12%), 4 isolates of *Escherichia coli* (11.12%), 3 isolates of *Klebsiella pneumoniae* (8.33%), 5 isolates of *Pseudomonas aeruginosa* (13.88%), 3 isolates of *Acinetobacter baumannii* (8.33%), 4 isolates of *Staphylococcus aureus* (11.12%), 3 isolates of *Staphylococcus epidermidis* (8.33%), 5 isolates of *Streptococcus agalactiae* (13.88%), 2 isolates of *Streptococcus pneumoniae* (5.55%), and 3 isolates *of Enterococcus faecium* (8.33%). The isolates showed the most sensitivity to imipenem and trimethoprim-sulfamethoxazole and the least sensitivity to erythromycin and tetracycline.

**Conclusions:**

The findings of the study indicate that sinusitis can contribute to fever of unknown origin in patients with cancer. Therefore, it is recommended to use a combination of molecular and phenotypic methods for accurate identification of isolates. This approach can provide more reliable and precise results, leading to better diagnosis and treatment of sinusitis infections in children with malignancy.

## Introduction

With the advancement of technology and medical sciences, there has been significant progress in controlling infectious diseases globally. However, factors such as AIDS, diabetes, cancers, and other immunodeficiency diseases have caused disruptions. In today's societies, the incidence of certain diseases and immune deficiencies is increasing steadily.^1^

Typically, malignancies are rare in children younger than age 15 years, accounting for only 1% to 3% of cases. However, it is evident that the pediatric population with cancer is at risk of developing a wide range of life-threatening infections. Leukemia is the most common type of cancer in children. It is well established in the literature that patients with cancer frequently develop neutropenia, which can increase their susceptibility to infections and mortality rates.^2^

The susceptibility of children with cancer to infections is heightened due to the cancer or the treatments they receive. The risk of infection is primarily linked to neutropenia.^3^ The frequent use of antimicrobial agents for various purposes, such as prevention, empiric therapy, prophylactic administration, specific or targeted therapy, and sometimes maintenance or suppressive therapy, can also influence the nature and range of infections that may occur, with particular concern for multidrug resistance. In patients with neutropenia, bacterial infections typically occur early, whereas fungal infections are less common during this phase.^4^

In malignancies, the presence of fever typically indicates an infection, although fever can also result from blood transfusions, thrombosis, and drugs. In some tumor types, fever can be a paraneoplastic syndrome caused by the malignancy itself. In these cases, the main source of the fever is unclear, leading to a phenomenon known as fever of unknown origin. This problem can pose a challenge in diagnosing the cause of fever, particularly when the underlying malignancy is difficult to treat. This creates a therapeutic challenge for the doctor.^5^ In patients with leukemia who have neutropenia, it is probable that more than 90% of cases of fever are the result of infections.^6^

Recognizing the pathogens that cause infections and comprehending the correlated risk factors are critical in creating effective approaches for diagnosing and treating patients with malignancies. Children with malignancies are vulnerable to various infections, including sinus infections. Sinusitis is primarily caused by bacterial infections, followed by fungal infections. The majority of these infections arise due to the reactivation of latent infections, particularly in patients with hematologic malignancies, cancer, and individuals who have had a transplant. Pathogenic bacteria such as *Moraxella catarrhalis, Streptococcus pneumoniae, Streptococcus pyogenes, Haemophilus influenza,* various species of *Staphylococcus*, anaerobic bacteria such as *Provetella, Fusobacterium*, and *Peptostreptococcus*, and gram-negative bacilli such as members of the Enterobacteriaceae family and *Pseudomonas* genus are the most common causes of sinusitis.^7^, ^8^, ^9^, ^10^, ^11^, ^12^, ^13^

Due to this concern, the present study aimed to evaluate the occurrence, diversity, and antibiotic susceptibility patterns of bacterial species that cause paranasal sinus infections in children with malignancy and unexplained fever. The study employs phenotypic and molecular methods to gain a better insight of the pathogenicity of these bacteria in patients with cancer.

## Methods and Materials

During a cross-sectional study from March 2022 to September 2022, a total of 90 samples of sinusitis secretions were collected from children with cancer aged 5 to 15 years who met the inclusion criteria. The samples were collected from Amir Kabir and Ayatollah Khansari hospitals in Arak City, Iran. Inclusion criteria for the study included age between 2 and 15 years, presence of malignancy, undergoing chemotherapy, fever for more than 3 days, and positive radiological signs of sinus infection. Exclusion criteria included age outside the specified range, absence of malignancy, absence of fever, absence of chemotherapy, and no radiological evidence of paranasal sinus infection.

Human research ethics approvals were received from the Khomein University of Medical Sciences Human Research Ethics Committee (IR.KHOMEIN.REC.1400.002). Furthermore, the study ensured that written informed consent was received from all patient or their guardians, and all methods used adhered to relevant guidelines and regulations. This indicates that the study was conducted in accordance with ethical principles and standards for human research.

Of the total 90 samples collected, 32 (35.55%) were from female patients and 58 (64.45%) were from male patients. The most common underlying disease among the patients was acute lymphocytic leukemia (ALL) with 47 samples (52.22%), followed by Burkitt's lymphoma with 17 samples (18.9%), aplastic anemia with 13 samples (14.45%), osteosarcoma with 7 samples (7.77%), and medulloblastoma with 6 samples (6.66%). Table 1 summarizes the sources and clinical details of the isolates.

**Table 1:** Sample characteristics, phenotypic characteristics, and genotypic characteristics of clinical isolates recovered from paranasal sinus infection in children with malignancy and fever of unknown origin.

No of isolates	Sample profile					Biochemical features													16S rRNA analysis		
	Designation	Gender	Age, y	Underlying disease	Polymicrobial	Gram stain	Catalase	Oxidase	Coagulase	MSA	Bile esculin	CAMP	Optochin	IMVIC	Urease	Lysozyme resistance	Decomposition of xanthene	Decomposition of Hypoxanthine	Similarity (%)*	Base pair difference†	Identification
1	AL1	Male	11	ALL	+	B+*	+	–	–	–	–	–	–	–	–	+	–	–	100	0/1010	*Nocardia cyriacigeorgica*
1	AL3	Male	7	Burkitt's lymphoma	+	B+	+	–	–	–	–	–	–	–	–	+	–	+	100	0/1010	*Nocardia nova*
2	AL2 and AL4	Female	3-8	ALL	-	B+	+	–	–	–	–	–	–	–	–	+	–	–	99.78	2/800	*Nocardia farcinica*
4	AL5, AL6, AL7 and AL8	Male/ female	8-14	Aplastic anemia, ALL	+	B-	+	–	–	–	–	–	–	++––	–	–	–	–	100	0/865	*Escherichia coli*
3	AL9, AL10 and AL11	Male/female	4-12	Osteosarcoma/ALL	–	B–	+	–	–	–	–	–	–	––++	+	–	–	–	99.9	1/923	*Klebsiella pneumoniae*
5	AL12, AL13, AL14, AL15 and AL16	Male/female	6- 14	Burkitt's lymphoma/aplastic anemia/ALL	+	B–	+	+	–	–	–	–	–	–––+	+	–	–	–	99.8	3/874	*Pseudomonas aeruginosa*
3	AL17, AL18 and AL19	Male	10-13	Medoloblastoma/Burkitt's lymphoma	–	B–	+	–	–	–	–	–	–	+––+	–	–	–	–	100	0/916	*Acinetobacter baumannii*
4	AL20, AL21, AL22 and AL23	Male/female	4-15	ALL/osteosarcoma/ Burkitt's lymphoma	+	C+	+	–	+	+	–	–	–	–	+	–	–	–	99.8	2/765	*Staphylococcus aureus*
3	AL24,AL25 and AL26	Male	6-12	ALL/aplastic anemia / medoloblastoma	–	C+	+	–	–	-	–	–	–	––++	+	–	–	–	100	0/894	*Staphylococcus epidermidis*
5	AL27, AL28, AL29, AL30 and AL31	Female	4-15	ALL/ osteosarcoma/ Burkitt's lymphoma	+	C+	–	–	–	–	–	+	–	–	–	–	–	–	99.8	2/763	*Streptococcus agalactiae*
2	AL32 and AL33	Male	7	ALL/osteosarcoma	–	C+	–	–	–	–	–	–	+	–	–	–	–	–	100	0/745	*Streptococcus pneumoniae*
3	AL34, AL35 and AL36	Male/female	9-13	Aplastic anemia/ Burkitt's lymphoma	+	C+	–	–	–	–	+	–	–	–	–	–	–	–	100	0/935	*Enterococcus faecium*

Al: designation of each isolate in study; ALL: acute lymphoblastic leukemia; CAMP: ChristieAtkinsMunch-Peterson; MSA: Manitol Salt Agar; rRNA: Ribosomal RNA.

⁎ Percentage similarity with the Type strain sequence.

† The difference between the number of sequence nucleotides and the number of sequence nucleotides of the Type strain.

### Isolation and conventional identification of isolated bacteria

The sampling and cultivation procedures followed standard methods for the isolation and identification of bacteria.^14^ Briefly, the patient was placed on his or her side, and a syringe connected to an irrigator was used to introduce physiological saline into the upper cavity of the nose. The resulting contents were collected and transferred to sterile containers with lids and transported to the laboratory. Under sterile conditions, the samples were centrifuged at 2000 × g for 10 minutes, and 100 µL sediment were cultured on blood agar, thioglycolate, MacConkey agar, and sothon agar, and incubated at 37°C for 4 weeks. A pure culture was then prepared from all the colonies obtained from the patient samples to perform diagnostic tests. For initial identification of bacteria, the following tests were conducted: pigment production, growth rate, nitrate reduction, urea, casein, starch, and growth in 0.4% gelatin, catalase, oxidase, Triple Sugar Iron (TSI), Indole, Methyl Red, Voges Proskauer and Citrate (IMVIC), Mannitol salt agar (MSA), hemolysis, and ChristieAtkinsMunch-Peterson (CAMP) test. Further identification was carried out using molecular tests.

### Molecular identification

The method described by Azadi et al^15^ was used to extract chromosomal DNA from actinomycetes isolates. To do this, a few colonies of bacteria grown on Sutton medium were added to 200 µL Tris EDTA (TE) buffer (1 mM EDTA, 10 mM Tris [pH 8]) and boiled for 15 minutes. Next, the microtube was placed in a –20°C freezer for 20 minutes, and this procedure was repeated twice. The suspension was then centrifuged at 8000 × g for 10 minutes, and the supernatant was transferred to a new microtube and centrifuged again at 13,000 × g for 20 minutes. The resulting pellet was resuspended in 50 µL TE buffer and stored at –20°C. The DNA was later resuspended in 50 µL Milli-Q water, Pars Banafsheh, IRAN and kept at –20°C.^16^

For species identification, polymerase chain reaction amplification and sequence analysis of an almost complete 16S ribosomal RNA (rRNA) gene were conducted, as described by Azadi et al.^15^ The sequencing was done by Pishgam Biotech Company (Tehran, Iran). The sequences were manually aligned and compared with sequences of closely related bacterial species obtained from the GenBank database using the jPhydit program version 1.1.3.^17^

### Drug Susceptibility Testing

The drug susceptibility testing (DST) for all isolates was performed using the Kirby- Bauer method on Mueller Hinton agar (MHA), following the Clinical & Laboratory Standards Institute (CLSI) 2021 criteria. MHA with 5% sheep blood was used for fastidious strains such as streptococci.^18^ The antibiotic discs used in this study were: trimethoprim/sulfamethoxazole (25 µg), erythromycin (15 µg), levofloxacin (5 µg), vancomycin (30 µg), clindamycin (2 µg), penicillin (10 µg), amoxicillin-clavulanic acid (30 µg), cefoxitin cefepime (30 µg), ceftazidim, cefotaxime (30 µg), ceftriaxone (30 µg), imipenem, tetracycline (30 µg), and clarithromycin (15 µg) (Mast; Merseyside, United Kingdom). The isolates were cultured with a sterile cotton swab on an 8-cm plate containing MHA medium. Then, antibiotic discs were positioned on the surface of the culture medium, with a standardized distance of 2.5 cm from 1 another. After 24 hours, the diameter of the zone of inhibition was measured and analyzed. The breakpoints for resistance and susceptibility were defined based on CLSI recommendations. Quality control of MICs was carried out by testing CLSI-recommended reference strains, including *Enterococcus faecalis* ATCC 29212 and *Staphylococcus aureus* ATCC 29213. Multidrug resistance was described as resistance to at least 3 different classes of antibiotics that were tested in this study.

### *Determination of methicillin-resistant* S aureus *isolates*

Methicillin-resistant *S aureus* (MRSA) isolates were characterized using an oxacillin agar screening test, which involved the use of MHA containing 4% sodium chloride and 2 µg/mL oxacillin. The growth of bacteria in the presence of oxacillin was considered a positive indication of MRSA strain.^19^ Two standard strains, including meticillin-sensitive *S aureus* ATCC 29213and MRSA ATCC 43300, were applied as negative and positive controls, respectively.

### Extended spectrum beta lactamases isolates

The identification of extended spectrum beta lactamase (ESBL) -producing isolates was carried out using a phenotypic test that followed the CLSI 2021 criteria. In this test, ceftazidime (30 µg) and a combination of ceftazidime with clavulanic acid (ceftazidime + clavulanic acid, 30/10 µg) discs were utilized. If the zone of inhibition around the combination discs was 5 mm greater than that around the ceftazidime disc alone, it was considered as a positive indication of ESBL production.^18^

### Sample size

Assuming that 40% of children had carriage of pathogenic bacteria in sinus, to detect the minimum sample size, the formula used was:

$$n=\frac{z^{2}\hat{p}\left(1-\hat{p}\right)}{d^{2}}$$

Where n = sample size, z = statistics corresponding to 95% level of confidence, $\hat{p}$ = expected prevalence (5%), and d = precision (5 %). Based on this formula the sample size required was 90.

### Statistical Analysis

The tests were performed in duplicate, and the average measures of samples were calculated to obtain the final result. Was calculated using the following formula: (number of positive samples / total sample size) × 100, and the results were presented as a percentage. The generated data in this study were presented in tables and percentages.

## Results

In the present study, 90 samples were collected in the following order from patients with malignancy and fever of unknown origin: 32 (35.55%) samples were collected from female patients and 58 (64.45%) samples were collected from male patients. The type of underlying disease in these people, in order of frequency, includes ALL 47 samples (52.22%), Burkitt's lymphoma 17 samples (18.9%), aplastic anemia 13 samples (14.45%), osteosarcoma 7 samples (7.77%), and medoloblastoma 6 samples (6.66%). The details of samples and isolates are presented in Table 1.

Among 90 samples of paranasal infections collected from patients with malignancy, based on phenotypic and molecular tests, 36 isolates (40%) of bacteria were isolated, including 4 isolates of *Nocardia* (11.12%), 4 isolates of *Escherichia coli* (11.12%), 3 isolates of *Klebsiella pneumoniae* (8.33%), 5 isolates of *Pseudomonas aeruginosa* (13.88%), 3 isolates of *Acinetobacter baumannii* (8.33%), 4 isolates of *S aureus* (11.12%), 3 of *Staphylococcus epidermidis* (8.33%), 5 isolates of *Streptococcus agalactiae* (13.88%), 2 isolates of *S pneumoniae* (5.55%), and 3 isolates of *Enterococcus faecium* (8.33%). Fungal species were isolated from the rest of the samples or no growth was observed, so they were excluded from the study. The specifications of the samples and the results of molecular and biochemical tests of isolates are given in Table 1.

The analysis of partial 16S rRNA gene sequences of the isolates revealed that specific nucleotide signatures were present for each genus. For *Nocardia* isolates, these signatures were observed at positions 70 to 98 (A–T), 307 (C), 293 to 304 (G-T), 614 to 626 (A–T), 631(G), 328 (T), 824e876 (T–A), 661 to 744 (G–C),825 to 875 (A–T), 843 (C), and 1122 to 1151 (A–T), and for gram-negative bacteria these signatures were observed at positions 70 to 98 (U–A), 843 (C), 1008 to 1021 (C–G), 139 to 224 (G–C), 1189 (C), 1308 to 1329 (C–G), and 1244 to 129 (C–G).

The isolates belonged to 10 genera and 12 validated species. The most prevalent bacterial species isolated in this study were: *P aeruginosa* and *S agalactiae* 5 isolates of each, *E coli* and *S aureus* 4 isolates of each, *K pneumoniae. A baumannii, S epidermidis*, and *E faecium* 3 isolates of each, *S pneumoniae* and *Nocardia cyriacigeorgica* 2 isolates of each, and *Nocardia farcinica* 1 isolate*.*

The phylogenetic relationship between our isolates and valid established species was demonstrated in a phylogenetic tree of the 16S rRNA gene, with a high bootstrap value, using the neighbor-joining method. The tree was generated using MEGA8 software, PA, USA, and it is depicted in the Figure.

**Fig:**
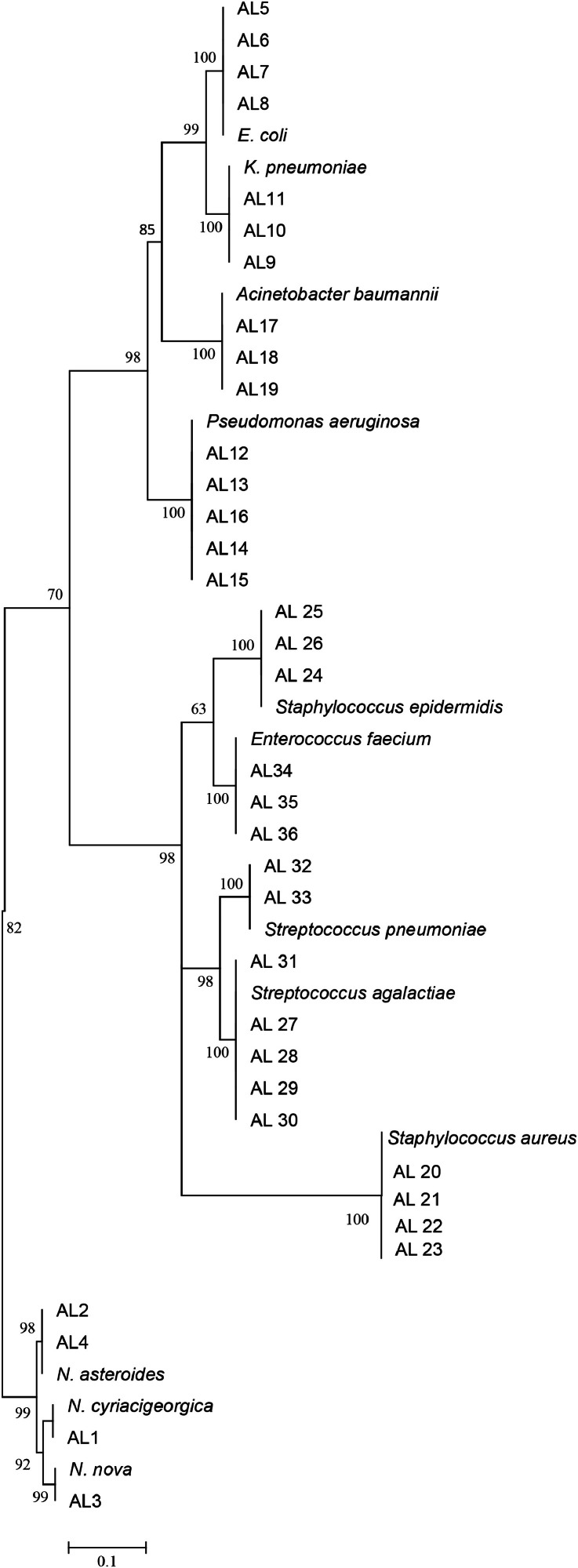
The 16S ribosomal RNA sequence-based phylogenetic tree for our clinical isolates and nearest standard species by using the neighbor-joining method depicted by MEGA8 software, PA, USA. At each node, bootstrapping values are represented.

### 16S rRNA gene sequence accession numbers

The GenBank accession numbers for the 16S rRNA gene sequences of isolated bacteria in this study are listed below, isolate AL1 *N cyriacigeorgica* (OQ195246), isolate AL2 *Nocardia asteroides* (OQ195247), isolate AL3 *Nocardia nova* (OQ195248), AL5 *E coli* (OQ195249), isolate AL11 *K pneumoniae* (OQ195250), isolate AL12 *P aeruginosa* (OQ195251), isolate AL17 *A baumannii* (OQ195252), isolate AL20 *S aureus* (OQ195254), isolate AL27 *S agalactiae* (OQ195255), isolate AL24 *S epidermidis* (OQ195256), isolate AL32 *S pneumoniae* (OQ195257), and isolate AL34 *E faecium* (OQ195258).

### DST results

The results of DST of all isolates based on CLSI standards showed that the isolates were most susceptible to imipenem and trimethoprim-sulfamethoxazole, and least susceptible to erythromycin, tetracycline, and clarithromycin. Additionally, the DST results for our isolates showed that 3 out of 4 isolates of *S aureus* were MRSA, 3 out of 5 isolates of *P aeruginosa* were multidrug resistant, 2 isolates of *K pneumoniae* were ESBL, and 1 out of 3 isolates of *A baumannii* was multidrug resistant. Detailed results of the DST are provided in Table 2.

**Table 2:** Drug susceptibility testing results of clinical isolates examined in the study based on disk diffusion method.

Isolate	Trimethoprim / Sulfamethoxazole	Erythromycin	Levofloxacin	Vancomycin	Clindamycin	Penicillin	Amoxicillin- clavulanic acid0	Cefepime	Ceftazidim	Cefotaxime	Ceftriaxone	Imipenem	Tetracycline	Clarithromycin	Drug susceptibility pattern
*Nocardia cyriacigeorgica*	S	S	S	–	S	–	R	S	–	S	S	S	S	R	
*Nocardia nova*	S	S	R	–	S	–	R	S	–	S	S	S	S	R	
*Nocardia astroides*	R	S	R	–	S	-	R	S	–	S	S	S	S	S	
*Escherichia coli*	S	R	I	–	R	–	S	I	S	–	R	S	R	–	
*Klebsiella pneumoniae*	S	I	S	–	I	–	S	R	R	–	R	S	S	–	ESBL
*Pseudomonas aeruginosa*	S	R	R	–	R	–	R	S	S	–	R	S	R	–	MDR
*Acinetobacter baumannii*	S	R	R	–	R	–	R	R	S	–	R	S	S	–	MDR
*Staphylococcus aureus*	R	R	R	S	R	R	S	–	–	S	S	–	R	R	MRSA
*Staphylococcus epidermidis*	S	R	R	S	S	S	S	–	–	S	S	–	R	R	
*Streptococcus agalactiae*	S	R	R	S	R	R	S	–	–	S	S	–	R	S	
*Streptococcus pneumoniae*	S	R	R	S	R	R	S	–	–	S	S	–	R	S	
*Enterococcus faecium*	R	R	R	S	R	R	S	–	–	S	S	–	S	S	

ESBL: Extended-spectrum beta-lactamases; I: Intermediate; MDR: multi-drug-resistant; MRSA: Methicillin-resistant S. aureus; R: Resistant; S. Susceptible.

## Discussion

Bacterial and fungal infections are significant causes of mortality in patients with cancer. According to published statistics, the death rate in patients with cancer in the absence of proper treatment has been reported as 42%. However, starting treatment on time can reduce complications and mortality. Fever and neutropenia are common side effects and significant causes of hospitalization in patients with immune system deficiency receiving immunosuppressive drugs, such as patients with cancer. Research shows that fever and neutropenia occur in 10% to 52% of patients with solid tumors and in 82% of people with leukemia. This common complication requires an average of 7 to 12 days of treatment and a cost of up to $1500, and is associated with an increased risk of death. Fever is the main indicator of infection in patients with cancer and malignancies. A fever of unknown origin, which is often seen in cancer patients, is defined as a temperature of more than 38.3 °C that lasts for more than 3 weeks and is caused by 3 days of hospitalization or 3 outpatient visits.^20^

Fever of unknown origin is most commonly caused by infections (30%–40%), neoplasms (20%–30%), connective tissue disease (20%), and miscellaneous causes (10%–20%). Among infectious agents, sinus infection is the most significant cause of unknown origin fever. Sinusitis refers to inflammation or swelling in the lining of the sinuses. Bacterial infections are the most common agent of sinusitis, followed by fungal infections. The majority of these cases arise due to the reactivation of latent infections, particularly in patients with hematologic malignancies, cancer, and individuals who have had a transplant. Reports of parasitic diseases and other unusual cases in these individuals are less common.^12^

In most reference laboratories, the identification of infectious bacteria is done by molecular methods such as 16S rRNA sequencing as a standard method. However, in Iran, due to the lack of advanced diagnostic facilities in most regional laboratories, many infections, especially sinusitis infections, are not diagnosed and antibiotic sensitivity is not determined.^21^ Therefore, based on the information provided, the aim of current study is screening, molecular identification, and antibiotic susceptibility pattern discernment of bacterial species isolated from paranasal sinus infections in patients with malignancy and unknown-origin fever by application of phenotypic, biochemical, and 16S rRNA gene sequence analysis DST as a standard procedure.

In the present study, 90 samples of sinusitis secretions were collected from children aged 5 to 15 years (average age = 9 years) with malignancy who met the inclusion criteria. The samples were obtained from the Amir Kabir and Ayatollah Khansari hospitals in Arak City and were analyzed for identification and antibiotic susceptibility pattern. Of the 90 samples, 32 (35.55%) were collected from female patients, and 58 (64.45%) were collected from male patients. The type of underlying disease in these people, in order of frequency, includes ALL 47 samples (52.22%), Burkitt's lymphoma 17 samples (18.9%), aplastic anemia 13 samples (14.45%), osteosarcoma 7 samples (7.77%), and medoloblastoma 6 samples (6.66%). Among 90 clinical samples, based on phenotypic and molecular tests, 36 isolates (40%) of bacteria were isolated, including 4 isolates of *Nocardia* (11.12%), 4 isolates of *E coli* (% 11/12), 3 isolates of *K pneumoniae* (8.33%), 5 isolates of *P aeruginosa* (13.88%), 3 isolates of *A baumannii* (8.33%), 4 isolates of *S aureus* (11.12%), 3 isolates of *S epidermidis* (8.33%), 5 isolates of *S agalactiae* (13.88%), 2 isolates of *S pneumoniae* (5.55%), and 3 isolates of *E faecium* (8.33%).

Our study revealed that the most of sinus infections in patients with cancer were associated with ALL and Burkitt's lymphoma. These findings are consistent with previous reports, including a study by Wang et al,^22^ in which most cases of sinusitis were observed in patients with types of leukemia such as ALL and lymphomas.

In the present study, bacterial infections were identified as the most common cause of fever in children with malignancy and fever of unknown origin, particularly sinusitis and urinary tract infections. These findings are consistent with other studies that have reported that infections (30%–40%) are the most common cause of fever of unknown origin in malignancies, including urinary tract infections and sinusitis.^23^^,^^24^

Based on the results of this study, it was found that the absolute number of neutrophils in the peripheral blood of patients with malignancy with fever of unknown origin was <500 per microliter. This finding is consistent with other studies, which have reported that when the absolute number of neutrophils in the peripheral blood is <500 per microliter, the patient is susceptible to various infections. When the neutrophil count drops below 200 per microliter, the possible risks become much more severe.^16^^,^^25^

In the present study, it was found that bacterial agents were the most common causes of sinusitis in children with malignancy, followed by fungal agents. These findings contradict other reports, including a report by Fung et al,^26^ and a report by Kurtbas et al^27^ in which the most common causes of sinusitis in children with malignancy were fungal agents followed by bacterial agents. Furthermore, in this study, the most common microbial agents isolated from the sinusitis infection of patients with malignancy were reported as *P aeruginosa, S agalactiae, E coli, S aureus, Nocardia, Acinetobacter,* and *S pneumoniae*. These results also confirmed other results in other parts of the world, in which the most common causes of sinusitis infection in malignancies were *S pneumoniae* and *S pyogenes, H influenzae*, staphylococci, and anaerobic bacteria such as *Provetella*, and gram-negative bacilli such as Enterobacteriaceae and *Pseudomonas*.^7^^,^^13^ In this study, 2 species of *N cyriacigeorgica and N farcinica* were isolated from sinusitis infections. This is the first report of these species being isolated from sinusitis infections and has not been previously published in scientific sources.

Our results, along with other findings, indicate that the complications of sinusitis in malignancies can be significant and vary depending on which sinus is infected. Among these complications, bone tissue destruction, subosseous abscess, meningitis, orbital cellulitis, asthma, cavernous sinus thrombosis, sleep disorders, osteomyelitis, smell disorders, and brain and dural abscesses can occur. These complications can be caused by direct diffusion, hematogenous spread, and septic thrombophlebitis.^28^

The results of the DST in our study revealed that the isolates analyzed were most sensitive to the antibiotics imipenem and trimethoprim-sulfamethoxazole, while showing the least sensitivity to the antibiotics erythromycin, ciprofloxacin, and clarithromycin. These findings are consistent with other studies related to the pattern of antibiotic resistance of infection-causing bacteria in hospitalized patients, indicating that most of the infectious agents are hospital infections and were transmitted to patients through the environment or hospital personnel. Furthermore, multidrug resistance, such as ESBL and MDR, especially in gram-negative species, was observed among the studied isolates, indicating the high genetic diversity and high resistance of the microbes that cause sinusitis infections in people with malignancy. This can lead to worsening of the condition and lack of effective treatment, resulting in prolonged hospitalization and increased health care costs.^29^

### Limitations

Among the limitations of this study is that some patients did not present to their physician during their period of fever and symptoms; for this reason, the sample size was just 90 sinus samples from children aged 5 to 15 years with malignancy. It is possible that if the sample size was larger, more accurate results would be obtained. Another limitation of this research is the difficulty in sampling and transferring the samples. Also, we could not sample all types of cancer.

## Conclusions

The present study identified bacterial agents that are the most common cause of sinusitis in children with malignancy, followed by fungal agents. Neutropenia was also found to be a significant risk factor for developing sinusitis infections in these patients. The complications of sinusitis infections in malignancies were found to be varied and dependent on which sinus is infected. These complications can be caused by direct diffusion, hematogenous spread, and septic thrombophlebitis. Therefore, early diagnosis and effective treatment are crucial to prevent these complications and improve patient outcomes. The DST analysis revealed multidrug resistance, particularly in gram-negative species, among the studied isolates. This highlights the importance of antibiotic stewardship and the need for effective infection control measures in hospital settings to prevent the spread of resistant strains. The findings of this study emphasize the need for increased awareness and vigilance in the diagnosis, treatment, and management of sinusitis infections in children with malignancy to improve patient outcomes and reduce the risk of complications.

## Declaration of competing interest

The authors have indicated that they have no conflicts of interest regarding the content of this article.
